# Absence of *Wolbachia* endobacteria in the human parasitic nematode *Dracunculus medinensis* and two related *Dracunculus* species infecting wildlife

**DOI:** 10.1186/1756-3305-7-140

**Published:** 2014-03-31

**Authors:** Jeremy M Foster, Frédéric Landmann, Louise Ford, Kelly L Johnston, Sarah C Elsasser, Albrecht I Schulte-Hostedde, Mark J Taylor, Barton E Slatko

**Affiliations:** 1 Parasitology Division, New England Biolabs, 240 County Road, Ipswich, MA 01938, USA; 2 Centre de Biochimie Macromoléculaire, CNRS, 1919 route de Mende, 34293 Montpellier Cedex 5, France; 3 Department of Parasitology, Liverpool School of Tropical Medicine, Pembroke Place, Liverpool L3 5QA, UK; 4 Department of Biology, Laurentian University, 935 Ramsey Lake Road, Sudbury, ON P3E 2C6, Canada

**Keywords:** *Dracunculus*, *Wolbachia*, Guinea worm

## Abstract

**Background:**

*Wolbachia* endosymbionts are a proven target for control of human disease caused by filarial nematodes. However, little is known about the occurrence of *Wolbachia* in taxa closely related to the superfamily Filarioidea. Our study addressed the status of *Wolbachia* presence in members of the superfamily Dracunculoidea by screening the human parasite *Dracunculus medinensis* and related species from wildlife for *Wolbachia*.

**Findings:**

*D. medinensis*, *D. lutrae* and *D. insignis* specimens were all negative for *Wolbachia* colonization by PCR screening for the *Wolbachia ftsZ*, 16S rRNA and *Wolbachia* surface protein (*wsp*) sequences. The quality and purity of the DNA preparations was confirmed by amplification of nematode 18S rRNA and cytochrome c oxidase subunit I sequences. Furthermore, *Wolbachia* endobacteria were not detected by whole mount fluorescence staining, or by immunohistochemistry using a *Wolbachia*-specific antiserum. In contrast, positive control *Brugia malayi* worms were shown to harbour *Wolbachia* by PCR, fluorescence staining and immunohistochemistry.

**Conclusions:**

Three examined species of *Dracunculus* showed no evidence of *Wolbachia* endobacteria. This supports that members of the superfamily Dracunculoidea are free of *Wolbachia*. Within the order Spirurida, these endosymbionts appear restricted to the Filarioidea.

## Findings

With the exception of *Loa loa*, all examined filarial nematodes that infect humans as their definitive host contain *Wolbachia*. These obligate intracellular symbionts have emerged as a novel target for filarial disease control [1-3]. Filarial species containing *Wolbachia* predominantly fall within the subfamilies Onchocercinae and Dirofilariinae of the family Onchocercidae, although *Madathamugadia hiepei* (subfamily Splendidofilariinae) is also colonized [4]. As a result, there has been extensive screening for *Wolbachia* in these taxa leading to the hypothesis that ancestral acquisition of *Wolbachia* occurred in the lineage leading to these subfamilies [5-7]. Screening for *Wolbachia* presence in phylogenetically divergent nematode species has consistently failed to identify these endobacteria [8,9], with the exception of an intriguing indication of a *Wolbachia*-like endosymbiont in the plant-parasitic Tylenchid nematode *Radopholus similis*[10,11]. However, apart from sampling within the Onchocercidae where *Wolbachia* are well known, there has been almost no screening of closely related families within the superfamily Filarioidea or within sister superfamilies within the order Spirurida.

*Dracunculus medinensis* is a member of the superfamily Dracunculoidea, a taxon closely related to the Filarioidea within the order Spirurida. Members of these two superfamilies have similar general morphology, are all tissue dwelling parasites, ovoviviparous, and use arthropod intermediate hosts. For these reasons, the two taxa are frequently discussed as a broader filarial nematode group [12]. However, *Dracunculus* species can be distinguished from true filarial nematodes by certain morphological features, molecular phylogenies and life cycle differences. For example, unlike filarial nematodes, the first stage larvae of *Dracunculus* species are expelled into the environment from where they can be ingested by non-haematophagous intermediate hosts (copepods). Infection of the mammalian host occurs after ingestion of the copepod and migration of third stage larvae through the intestinal wall as opposed to filarial transmission via blood feeding of the arthropod host. *D. medinensis*, perhaps the longest nematode infecting humans, was until recently a major cause of human morbidity infecting ~3.5 million individuals in Africa and Asia. Despite tremendous progress towards eradication of *D. medinensis* by the global Guinea Worm Eradication Program, dracunculiasis persists in 4 African nations due, in part, to resource limitations, political instability and civil war [13-17]. Although prospects for complete eradication remain promising, positive identification of *Wolbachia* endobacteria in *D. medinensis* would offer another reagent in the elimination toolbox. Targeting *Wolbachia* with doxycycline is validated as a control method for human filariasis and is a therapy particularly well suited to individual treatment rather than mass drug administration. Delivering doxycycline to the ~500 remaining dracunculiasis patients would be a realistic goal and assist in containment of the disease since a long-lasting sterility of filarial nematodes is an early consequence of antibiotic treatment [3].

We undertook screening for the possible presence of *Wolbachia* in the human pathogen *D. medinensis* and in *D. lutrae* and *D. insignis* recovered from wildlife since they are representatives of the Dracunculoidea, a taxonomic group for which no information on the occurrence of *Wolbachia* endosymbionts is available.

### Parasite specimens and DNA extraction

Three sections of female *D. medinensis* obtained from different specimens recovered from human infections in Ghana were provided by Dr Mark Eberhard, Centers for Disease Control and Prevention, Athens, GA. Specimens of *D. lutrae* and *D. insignis* were recovered from otter (*Lontra canadensis*) and mink (*Neovison vison*), respectively in Ontario, Canada as described [18]. The parasite material was stored in ethanol at -20°C prior to extraction of genomic DNA by standard procedures.

### PCR and DNA sequencing

The suitability of the extracted gDNA for PCR was examined by attempted amplification of part of the nematode 18S rRNA gene. Primers Drac18Sf 5′-ACTGGAGGAGGAATCCAACGTGCTATGT-3′ and Drac18Sr 5′-TGTGTACAAAGGGCAGGGACGTAA-3′ were designed based on 18S rRNA sequences of *D. medinensis*, *D. lutrae* and *D. insignis* [GenBank: AY947720, GenBank: JF934737, GenBank: AY947719]. PCR reactions (25 μl) used Q5 High-Fidelity 2X Master Mix (New England Biolabs) with 0.5 μM each primer and ~50 ng DNA. Cycling consisted of one cycle of 98°C for 1.5 min, followed by 30 cycles of 98°C, 10 s; 71°C, 20 s; 72°C, 20 s, then a final extension at 72°C for 2 min. The PCR products were cloned into the SmaI site of pUC19 (New England Biolabs) and sequenced on both strands using a 3730xl DNA Analyzer (Applied Biosystems). Identical 166 bp sequences were obtained which matched exactly the 18S rRNA sequences from these 3 species available in the NCBI database. Additional Barcode of Life primers for cytochrome c oxidase subunit 1 (COI) [19] were redesigned based on the *D. medinensis* mitochondrial genome sequence [GenBank: JN555591]. PCR, cloning and sequencing were as described for 18S rRNA, but PCR used primers DracCOIf 5′-AAAGGACTAATCATAAGGATATTGG-3′ and DracCOIr 5′-TAAACCTCAGGATGACCAAAAAATCA-3′ and a 61°C annealing temperature. Distinct 655 bp sequences were obtained from each species, which matched respective COI sequences in the NCBI database [GenBank: EU646545, GenBank: EU646601, GenBank: HQ216219] at ≥ 99% nucleotide identity. This confirmed the correct identification of each *Dracunculus* species, which was necessary since both *D. insignis* and *D. lutrae* infect the otter [18].

The possible presence of *Wolbachia* was examined by screening for the *Wolbachia* 16S rRNA gene and the genes encoding FtsZ and Wsp (*Wolbachia* surface protein). These genes are routinely used for *Wolbachia* identification and phylogenetic analyses [5,20,21]. The primers for 16S rRNA (16SwolbF, 16SwolbR3), *ftsZ* (ftsZ357F, ftsZ788R) and *wsp* (wsp81F, wsp691R) have been detailed elsewhere [22]. PCR was performed as described above for nematode 18S rRNA except that the annealing temperatures were 57°C for *wsp* and 61°C for 16S rRNA and *ftsZ*. No PCR products were obtained from any of the *Dracunculus* DNA samples when attempting amplification of these three *Wolbachia* sequences. In contrast, the correct amplicons were generated from *B. malayi* DNA (data not shown).

### Whole mount fluorescence staining

**Figure 1:**
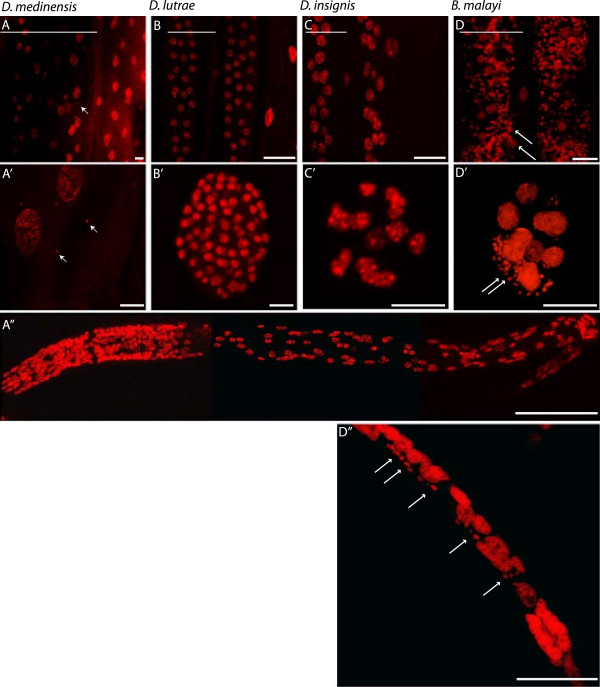
**Cellular analysis indicates absence of endosymbionts in *Dracunculus *species.** Tissues from *D. medinensis***(A)**, *D. lutrae***(B)** and *D. insignis***(C)** were stained with propidium iodide as described [23] and compared to *Wolbachia*-harbouring *Brugia malayi* tissues **(D). A**, **A’**, **B**, **D** and **C**, lateral hypodermal cords (scale bar = 50 μm); **B’**, **C’** and **D’**, embryos (scale bar = 10μm); **A”** and **D”**, microfilariae (scale bar = 50 μm). The bars at top left of the panels represent the length of half a cord. In panels **A** and **A’**, arrows point to smaller nuclei of about 4 μm, from tissue underlying the hypodermis. In panels **D**, **D’** and **D”**, long arrows point to *Wolbachia* foci.

Portions of female worms of all three *Dracunculus* species were examined for *Wolbachia* presence by whole mount fluorescence staining. Female *B. malayi* were used as a positive control. Worms were fixed, treated with RNase A (Sigma) at 10 mg/ml in PBS, and their DNA stained using propidium iodide (Molecular Probes), then subsequently imaged as described previously [23]. *Wolbachia* were not detected in either the lateral cords or embryos/microfilariae of any *Dracunculus* species (Figure 1). A few punctate dots of staining were observed in the lateral cord region of *D. medinensis* (Figure 1, Panels A, A’) but these were approximately 4 μm in diameter and, therefore, considerably larger than *Wolbachia*. We believe these are nuclei of nematode cells in tissue lying beneath the hypodermis. In contrast, abundant *Wolbachia* were observed in both the cords and embryos of *B. malayi* (Figure 1, Panels D, D’).

### Immunohistochemistry

**Figure 2:**
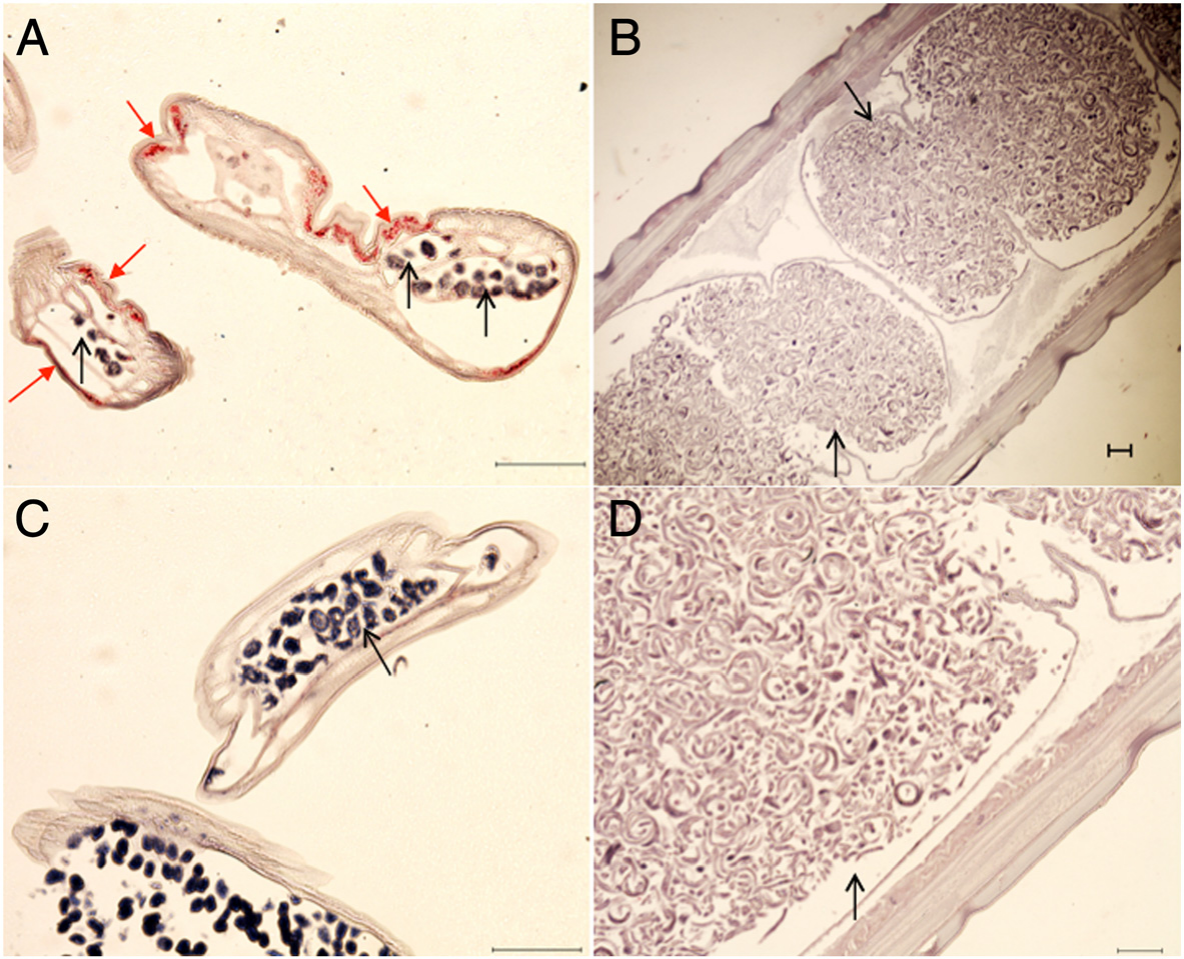
**Anti-WSP staining in transverse sections of *D. medinensis *and *B. malayi*. ***Wolbachia* are shown as red punctate staining in the lateral hypodermal cords of *B. malayi***(A)** but are absent from *D. medinensis***(B and D)** as well as in tetracycline-treated *B. malayi***(C)**. Solid red arrows highlight *Wolbachia* within the hypodermal cords. Open black arrows indicate uteri containing developing embryos. *B. malayi* images are x200 magnification. *D. medinensis* images are x40 **(B)** and x100 magnification **(D)**. Scale bars = 100 μm.

Segments of female *D. medinensis* were prepared for sectioning and immunohistochemistry using a rabbit polyclonal antiserum raised to WSP as described previously [22]. *B. malayi* served as a positive control and tetracycline-treated *B. malayi* (*Wolbachia*-depleted) were used in comparison to show the specificity of the anti-WSP serum. Sections of *D. medinensis* consistently showed no anti-WSP staining (Figure 2). In contrast, abundant staining was observed in the lateral cords of the *B. malayi* positive control. Staining in *B. malayi* was not observed when worms previously exposed to a 6 week tetracycline treatment to deplete their endosymbionts were examined. This differential staining of tetracycline-treated and untreated *B. malayi* proved that the positive staining is due to *Wolbachia*.

In conclusion, we screened the human parasitic nematode *D. medinensis* as well as *D. lutrae* and *D. insignis* recovered from Canadian wildlife for the presence of *Wolbachia* endosymbionts. All three species were negative for each of three *Wolbachia* sequences screened for by PCR using primer pairs previously used for *Wolbachia* screening of filarial nematodes [5,21,22,24]. Notably, the *wsp* and 16S rRNA primer pairs have previously been used to amplify corresponding sequences from more divergent *Wolbachia* strains that infect arthropods [24,25], implying that our inability to amplify from *Dracunculus* samples was not due to sequence divergence. Amplification of *Dracunculus* 18S rRNA and COI sequences demonstrated the suitability of the DNA preparations for use in PCR and confirmed correct identity of the three species.

We were unable to detect *Wolbachia* in the three *Dracunculus* species by whole mount fluorescence staining, or in *D. medinensis* by immunohistochemistry using an anti-WSP serum that cross-reacts with *Wolbachia* from diverse filarial nematodes and *Aedes albopictus* mosquitos [2,26,27]. In contrast, in all experiments, our positive control *B. malayi* gave clear evidence of *Wolbachia* infection. The correct PCR amplicons were generated from *B. malay*i DNA and *Wolbachia* were readily visualized by both whole mount fluorescence and immunohistochemistry, indicating that the reagents and methodologies used were appropriate.

Our finding that three species of *Dracunculus* lack detectable *Wolbachia* supports that all members of the genus lack these endobacteria and precludes use of doxycycline as an additional tool in the efforts to finally eradicate human dracunculiasis. This is the first report of *Wolbachia* screening in the superfamily Dracunculoidea. The apparent lack of *Wolbachia* infection in members of this taxon is consistent with the notion that within the order Spirurida these endosymbionts are restricted to the superfamily Filarioidea and, more specifically, the family Onchocercidae [5,7].

## Competing interests

The authors declare that they have no competing interests.

## Authors’ contributions

JF, MT and BS conceived and designed the study and wrote the manuscript. SE and AS collected samples and identified species. JF, FL, LF and KJ performed the experiments. FL, LF and KJ generated the figures. All authors contributed text and read and approved the final manuscript.
